# Diagnosis of Antipsychotic Medication‐Induced Severe Hypertriglyceridaemia by the Phenomenon of Pseudohyponatraemia and Successful Management With Variable‐Rate Intravenous Insulin Infusion: A Case Report

**DOI:** 10.1155/crie/2376136

**Published:** 2026-09-08

**Authors:** Adam Coleman, Grace Kettyle, Monica Monaghan, Edward McKeever

**Affiliations:** ^1^ Department of Cardiology, South West Acute Hospital, Enniskillen, UK; ^2^ Department of Endocrinology, South West Acute Hospital, Enniskillen, UK

**Keywords:** antipsychotic, case report, hypertriglyceridaemia, insulin infusion, pseudohyponatraemia

## Abstract

**Background:**

Hypertriglyceridaemia (HTG) is a common condition resulting from genetic and environmental factors. Second‐generation antipsychotic (SGA) medications are classically associated with the development of metabolic syndrome, of which HTG is a hallmark feature. Severe HTG is the third most common cause of acute pancreatitis, which has significant associated morbidity and mortality risk. It is therefore vital to rapidly and sustainably reduce triglyceride levels in patients presenting with severe HTG.

**Clinical Case:**

A 35‐year‐old gentleman with a history of psychiatric illness taking SGA medications (quetiapine and risperidone) presented to our hospital with apparent hyponatraemia. With associated hyperosmolality (302 mOsm/kg), a diagnosis of pseudohyponatraemia was made. Upon further investigation, pseudohyponatraemia was identified to be due to severe HTG (72.8 mmol/L) and hyperglycaemia (26.0 mmol/L). There were no clinical or biochemical features of pancreatitis. A variable‐rate infusion of intravenous insulin was commenced. Over the 10‐day admission a fall in triglycerides to 10.5 mmol/L was observed without any adverse events. Acute pancreatitis did not develop during the inpatient admission or during outpatient follow‐up, and triglyceride levels have fallen substantially (3.2 mmol/L at 5 months) having been switched from quetiapine and risperidone to aripiprazole, an SGA associated with less metabolic disturbance.

**Conclusion:**

This case demonstrates the safe and effective management of SGA‐associated severe HTG with intravenous insulin infusion to prevent acute pancreatitis. It also highlights the utility of pseudohyponatraemia as a useful way of detecting severe HTG and the impact of SGA medications in the aetiology of severe HTG.

## 1. Introduction

Hypertriglyceridaemia (HTG) is an extremely common finding worldwide, affecting up to 30% of adults [1]. The causes of HTG are academically divided into primary (genetic) and secondary (acquired) factors; however, there is a complex interaction between these factors that leads to the development of HTG, which is reflected by the significant ethnic and regional variability in the prevalence. Medications are common contributors to HTG, with antipsychotics being one of the most commonly implicated [1, 2]. Whilst second‐generation ‘atypical’ antipsychotics have been revolutionary in their reduced risk of producing extrapyramidal side effects compared to their first‐generation counterparts, they are strongly associated with metabolic syndrome as manifested by weight gain, increased risk of type 2 diabetes mellitus, and dyslipidaemia [2, 3]. This is compounded by the fact that patients who require antipsychotic medications for their primary indications of psychosis or schizophrenia are already at an increased risk of developing metabolic syndrome independent of antipsychotic medication [4]. HTG is an important finding to identify and manage, both directly and indirectly, for its association with cardiovascular disease and also as a cause of acute pancreatitis [5, 6].

## 2. Case Presentation

A 35‐year‐old male with a diagnosis of schizoaffective disorder and autism spectrum disorder, managed since late adolescence with multiple antipsychotic and mood‐stabilising agents, was referred following routine primary care bloods showing severe hyponatraemia (serum sodium 124 mmol/L). He reported no symptoms, and there was no recent change in mental or physical state. He was assessed and managed by the Department of Endocrinology at South West Acute Hospital, Enniskillen, UK, during a 10‐day inpatient admission in March 2024, with outpatient follow‐up over the subsequent 5 months.

Physical examination revealed a BMI of 41.8 kg/m^2^, and he exhibited marked central obesity and moderate acanthosis nigricans over the neck and axillae, reflecting chronic insulin resistance. The patient appeared euvolaemic, with no evidence of fluid overload, dehydration or acute illness. He denied any gastrointestinal symptoms, including abdominal pain, nausea, vomiting or steatorrhoea. Renal function was normal (eGFR >60 mL/min/1.73 m^2^ and creatinine 55 μmol/L), as were baseline liver indices.

Historical data showed a notable weight gain over 17 years (70 → 130 kg, BMI class III). At age 21, he had a prior admission for diabetic ketoacidosis requiring insulin therapy, which was later tapered with normalisation of glucose tolerance following modification of the antipsychotic regimen and institution of statin therapy for dyslipidaemia. At the time of presentation, his medications were quetiapine 800 mg daily, risperidone 2 mg nightly, lithium 1 g daily and lamotrigine 100 mg daily, with atorvastatin continued for hyperlipidaemia.

Emergency department biochemical analysis confirmed persistently depressed sodium (124 mmol/L), with concurrent elevated serum osmolality (302 mOsm/kg, reference 275–295 mOsm/kg), hyperglycaemia (glucose 26.0 mmol/L, reference 4–7.7 mmol/L), and HbA1c of 109 mmol/mol. When the sodium level was corrected for hyperglycaemia using the Katz [7] formula, hyponatraemia persisted with an adjusted sodium of 130 mmol/L (reference 135–146 mmol/L). Most notably, his triglycerides were measured to be 72.8 mmol/L (reference 0.4–1.7 mmol/L). The lipid panel also revealed elevated total cholesterol at 9.2 mmol/L and low HDL at 0.94 mmol/L. Despite the severe HTG, there were no clinical or biochemical features of acute pancreatitis; serum amylase was normal, liver enzymes were normal, and there was a complete absence of systemic inflammatory response or haemodynamic instability [5, 8].

Given the lipaemic nature of the sample and a flag from laboratory staff, point‐of‐care sodium testing was performed using the Roche Cobas B 123 POC system. This measurement confirmed that the laboratory hyponatraemia was artefactual (pseudohyponatraemia), as the true sodium level was 137.3 mmol/L (reference: 135–145 mmol/L).

Initial management included the commencement of subcutaneous insulin (Novomix 30 twice daily) and metformin 500 mg twice daily. Following interdisciplinary consultation (including endocrinology, psychiatry, clinical biochemistry and pharmacy), the decision was made to initiate an inpatient variable‐rate intravenous insulin infusion (VRIII) based on local diabetes protocols and published evidence supporting insulin therapy in severe HTG [9, 10].

For a body weight of ~130 kg, published fixed‐rate intravenous insulin regimens for severe HTG typically fall in the range of 0.07–0.10 units/kg/h, equivalent to roughly 9–13 units/h in this patient [9]. Because there was no clinical or biochemical evidence of acute pancreatitis, no organ dysfunction, and no haemodynamic instability, we elected to use a more conservative, glucose‐titrated VRIII regimen rather than an upfront fixed‐rate weight‐based insulin infusion.

Dietary consultation recommended portion‐controlled meals emphasising non‐starchy vegetables and the avoidance of refined sugars or high‐fat foods. Since there was no evidence of acute pancreatitis, there was no need for fasting, enteral or parenteral nutrition.

In severe HTG complicated by pancreatitis, fasting or a very low‐fat diet is commonly recommended to reduce chylomicron formation and pancreatic stimulation [11, 12]. In this case, however, the patient had no abdominal pain, normal pancreatic enzymes and no inflammatory response and remained clinically stable throughout admission. We therefore opted against fasting and instead advised portion‐controlled meals, avoidance of sugar‐sweetened drinks, restriction of refined carbohydrates, and avoidance of visibly high‐fat foods such as fried foods and cream‐based meals. This more liberal approach allowed treatment under real‐world post‐prandial conditions while still targeting the major dietary drivers of triglyceride elevation.

Pharmacological management for the HTG was decided by consensus, involving the addition of fenofibrate and omega‐3 fatty acids alongside an uptitration of atorvastatin to 40 mg daily.

Serial capillary glucose readings guided insulin infusion, which was started at 0 units/h for glucose <4.0 mmol/L and for 4.0–6.6 mmol/L, then 1, 2, 3, 4 and 6 units/h for readings of 6.7–9.9 mmol/L, 10−13.3 mmol/L, 13.4–16.6 mmol/L, 16.7–19.9 mmol/L and ≥20.0 mmol/L, respectively. This initial regimen was deliberately conservative, prioritising safety and reducing the likelihood of hypoglycaemia in a patient with marked metabolic derangement but without critical illness. Following a multidisciplinary review on day 5, the regimen was intensified to a maximum of 12 Units/h, with 0, 1, 2, 4, 6, 8 and 12 units/h for the same glucose bands. This escalation corresponded to ~0.09 units/kg/h at peak delivery, thereby approaching the lower end of published weight‐based fixed‐rate regimens while preserving the practical advantages of a nurse‐led variable‐rate protocol [9]. All blood glucose, triglyceride, and sodium levels were monitored twice daily.

By day 10, the patient’s triglycerides had declined to 10.5 mmol/L, and there was marked improvement in blood glucose (average 7.4 mmol/L) with pseudonormalisation of sodium levels. No adverse events occurred. The absence of hypoglycaemia or electrolyte‐related complications supports the safety of this stepwise escalation strategy in clinically stable severe HTG. VRIII was stopped, and the patient was maintained on subcutaneous insulin alongside metformin. The daily triglyceride response across the admission is shown in Table 1, and the initial and intensified VRII regimes are shown in Table 2.

**Table 1:** Fall in serum triglyceride concentration from admission (day 1) to discharge (day 10) following commencement of a variable‐rate intravenous insulin infusion (VRIII) regimen with intensified regimen from day 5.

Day of admission	Serum triglycerides (mmol/L)
1	72.8
2	Not measured
3	47.4
4	41.1
5	32.4
6	22.4
7	Not measured
8	20.0
9	17.4
10	10.5

*Note:* Triglycerides were not measured on days 2 and 7.

Abbreviation: VRIII, variable‐rate intravenous insulin infusion.

**Table 2:** Initial and intensified variable‐rate intravenous insulin infusion (VRIII) regimens.

Capillary blood glucose (mmol/L)	Initial regimen, days 1–4 (units/h)	Intensified regimen, days 5–10 (units/h)
<4.0	0	0
4.0–6.6	0	1
6.7–9.9	1	2
10.0–13.3	2	4
13.4–16.6	3	6
16.7–19.9	4	8
≥20.0	6	12

*Note:* The initial regimen was used from day 1 to day 4. Following multidisciplinary review the regimen was intensified from day 5 until discharge on day 10, corresponding to ~0.09 units/kg/h at peak delivery for a body weight of 130 kg. Infusion rate was titrated against hourly capillary blood glucose.

Abbreviation: VRIII, variable‐rate intravenous insulin infusion.

Psychiatric supervision initiated a cross‐tapering of the high‐metabolic‐risk agents, quetiapine and risperidone, with aripiprazole [13]. Lithium and lamotrigine doses were maintained for mood stabilisation.

On discharge (10 days after admission), the patient was maintained on subcutaneous insulin alongside metformin. 5 months later, the patient’s triglyceride level was 3.2 mmol/L, HbA1c had declined to 64 mmol/mol, and sodium remained in the normal range on repeat indirect ion‐selective electrode (iISE) measurement. No recurrence of laboratory artefacts or clinical deterioration was observed, and statin therapy was continued without intolerance.

## 3. Discussion

This case illustrates the complex interaction between psychiatric medication, metabolic syndrome, and biochemical artefacts that can significantly impact the diagnostic accuracy and therapeutic strategy. The importance of a comprehensive approach, spanning clinical evaluation, laboratory methodology awareness, collaborative interdisciplinary management and judicious pharmacological intervention, cannot be understated.

### 3.1. Pseudohyponatraemia and Laboratory Methodology

Hyponatraemia is a common electrolyte disturbance, but its significance depends critically on the method by which sodium is measured and the presence of confounding variables such as high glucose, protein or lipid concentrations [8, 14]. Most hospital laboratories use the iISE method. This method involves diluting a small volume of serum with a standard solution, followed by mathematical processing to calculate the final sodium concentration. Crucially, this method and its calculations rely on the assumption that serum is composed of normal proportions of serum water (93%) and serum solids, that is, lipids and proteins (7%). When these proportions are abnormal and serum solids are relatively increased—such as in severe HTG—the serum water content is relatively decreased. Consequently, the indirect method produces an inaccurately low sodium result because the mathematical assumption of ‘normal’ serum proportions no longer holds [8, 14].

In contrast, direct ISE assays (often found in point‐of‐care blood gas analysers) measure sodium concentration in undiluted whole blood/serum samples. Because no dilution occurs, there is no assumption‐based mathematical processing of the measurement, and the result remains accurate regardless of lipid or protein levels. Direct ISE is therefore preferred in cases where an abnormal serum solid proportion is expected or suspected, for example, in severe HTG or paraproteinaemia [8].

In this case, both hyperglycaemia and HTG co‐existed, with each contributing to the pseudohyponatraemia observed. Using the widely used Katz [7] formula to correct serum sodium for the degree of hyperglycaemia, an adjusted sodium of 130 mmol/L is produced. When this adjusted result is then further adjusted for the degree of HTG, using the formula established by Dimeski et al. [15], a final (indirect) sodium of 135.6 mmol/L is produced, which more closely approximates the point‐of‐care analyser (direct) result of 137.3 mmol/L.

Interestingly, even though pseudohyponatraemia is an inaccuracy, it does have diagnostic utility. In this patient, it acted as a ‘smoke signal’ for the underlying pathology that could give rise to it. Without this laboratory artefact, it is unlikely that a lipid profile would have been checked so early in the admission, potentially delaying the diagnosis and treatment of severe HTG. More broadly, it is important for clinicians to have an awareness of lab artefacts such as pseudohyponatraemia in order to prevent unnecessary investigation and inappropriate treatment for ‘hyponatraemia’, which, if managed with hypertonic saline or fluid restriction, could result in adverse outcomes [14]. Calculation of corrected sodium using established formulas and consideration of true osmolality must be routine in endocrinology and acute medicine.

### 3.2. Antipsychotic Medication and Metabolic Risk

Second‐generation antipsychotics (SGAs) have transformed psychiatric management but are strongly associated with metabolic syndrome, including obesity, insulin resistance, glucose intolerance and dyslipidaemia [2, 3]. Clozapine, olanzapine, quetiapine and risperidone carry higher risk profiles, whereas aripiprazole and lurasidone are associated with lower metabolic complications [13]. Mechanistically, SGAs exert antagonism or partial agonism at dopamine D2 and serotonin 5‐HT2A receptors, influencing appetite, glucose utilisation and lipid homeostasis [3, 16].

The cumulative effect in predisposed individuals, such as those with baseline obesity or central adiposity, can provoke rapid metabolic deterioration, sometimes only apparent when laboratory artefacts—such as pseudohyponatraemia—are identified on routine monitoring [4]. In addition, psychiatric patients are less likely to access routine metabolic care, amplifying morbidity [4, 16].

### 3.3. Management of Severe HTG

Severe HTG is a potent trigger for acute pancreatitis, especially at levels above 11 mmol/L, with direct risk for organ failure, systemic inflammatory response and mortality [5, 6]. In the absence of pancreatitis, as in this case, therapy must still aim for a rapid reduction of triglyceride levels to prevent acute complications. Evidence supports variable‐rate or fixed‐rate insulin infusion regimens, commonly around 0.07–0.10 units/kg/h, titrated to glycaemic response and patient safety [9]. Insulin accelerates clearance of chylomicrons and VLDL by upregulating lipoprotein lipase, facilitating triglyceride catabolism [17].

Our use of a conservative, glucose‐titrated VRIII protocol that was subsequently escalated towards standard weight‐based doses illustrates a pragmatic approach in non‐critical severe HTG, balancing the need for rapid triglyceride reduction against the risks of hypoglycaemia and rapid osmotic shifts. This is particularly relevant where formal HTG‐specific insulin protocols are unavailable and clinicians must adapt existing diabetes VRIII pathways to an uncommon metabolic emergency [9].

Dietary fat restriction is a cornerstone of severe HTG management, with many authors advocating very low‐fat diets or temporary fasting to reduce chylomicron production, particularly in the setting of acute pancreatitis [11, 12]. In contrast, we adopted a more liberal, portion‐controlled regimen in a clinically stable patient without pancreatitis, highlighting that insulin‐based therapy can be effective even under real‐world post‐prandial conditions, though more restrictive fat reduction is likely warranted when pancreatitis or organ dysfunction is present [11, 12].

Alternative therapies—plasmapheresis, fibrates, EPA, niacin, and omega‐3 fatty acids—are indicated in refractory cases or where insulin therapy is contraindicated. Plasmapheresis offers rapid triglyceride reduction but at considerable procedural risk, cost, and need for central venous access, thus being reserved for life‐threatening cases [10, 18].

### 3.4. Ongoing Management and Psychiatric Considerations

The central focus of longer‐term management in SGA‐treated patients is risk mitigation. Switching to lower metabolic risk agents (e.g., aripiprazole), regular monitoring of lipids and glucose and lifestyle interventions—including weight management, physical activity and dietary counselling—are essential components, though often logistically difficult to implement [13, 16].

Although aripiprazole carries a lower metabolic risk, rare cases of true hyponatraemia via increased AVP secretion have been described, mandating continued vigilance for electrolyte disturbances [19]. This case underscores the importance of sustained collaboration between psychiatry, endocrinology and clinical nutrition to optimise psychotropic regimens and cardiometabolic risk over time.

## 4. Conclusion

Pseudohyponatraemia, revealed during routine laboratory assessment in an SGA‐treated psychiatric patient, was the key diagnostic clue for severe HTG and the underlying metabolic syndrome. A targeted, variable‐rate intravenous insulin regimen achieved safe, effective triglyceride reduction and sodium normalisation, with further improvement following transition to lower metabolic risk SGA therapy and statin up‐titration. Detailed recognition of laboratory artefacts, interdisciplinary cooperation, and a holistic approach to metabolic syndrome management are essential for optimal clinical outcomes in similar cases.

## Author Contributions

**Adam Coleman**: conceptualisation, data collection, writing – original draft. **Grace Kettyle**: data collection, writing – review and editing. **Monica Monaghan**: clinical supervision, writing – review and editing. **Edward McKeever**: conceptualisation, clinical supervision, writing – review and editing, manuscript guarantor.

## Funding

The authors received no specific funding for this work.

## Disclosure

All authors have read and approved the final version of the manuscript. Edward McKeever had full access to all the data in this study and takes complete responsibility for the integrity of the data and the accuracy of the data analysis.

## Ethics Statement

This case report was conducted in accordance with the Declaration of Helsinki (1964) and its later amendments (formal ethics approval was not required for this single retrospective case report at the Western Health and Social Care Trust).

## Consent

Written informed consent for the publication of this case report, including all clinical and biochemical details contained herein, was obtained from the patient. The signed consent form is securely archived by the corresponding author and is available to the journal or the relevant ethics committee on request.

## Conflicts of Interest

The authors declare no conflicts of interest.

## Data Availability

The data supporting the findings of this case report are contained within the article. Further anonymised clinical data are available from the corresponding author upon reasonable request, subject to the constraints of patient confidentiality and institutional information governance approval.
